# A bio-environmental perspective on Emirati female college students’ experiences in virtual learning communities of inquiry

**DOI:** 10.1186/s41239-021-00281-y

**Published:** 2021-08-26

**Authors:** Aysha Saeed AlShamsi

**Affiliations:** Faculty of Education, Department of Early Childhood Education, Higher Colleges of Technology, Al Ain, P.O. Box 20711, 25026 Abu Dhabi, United Arab Emirates

**Keywords:** Bio-ecology perspective, Cognitive skills, Community of Inquiry, Convergent parallel mixed methods, Interpretive phenomenology, Online classes, Postsecondary education, Virtual classrooms

## Abstract

During the coronavirus pandemic, educational institutions were forced to shift to virtual learning. Drawing on the Community of Inquiry framework and bioecological perspective, this research explores the virtual learning experiences of female college students at one higher education institution in the United Arab Emirates using an interpretive phenomenological paradigm. A convergent parallel mixed method design was implemented with participants (*N* = 350) who completed a questionnaire about the challenges of virtual learning followed by semi-structured interviews (*N* = 10). Observations, journals, and peer-reviewed literature was also used to explore the influence of cognitive, social, and teaching presence on students’ perceptions. The data were analyzed using descriptive statistics and thematic analysis. The researcher found students had a high perception of the three influences of Community of Inquiry framework and were aware of its importance. Furthermore, there were clear relationships between cognitive and teaching presence and cognitive and social presence. The importance of online teaching and learning strategies supports the interactivity of these presences.

## Introduction

In response to the coronavirus (COVID-19) threat, higher education institutions in the United Arab Emirates (UAE), like others across the world, cancelled face-to-face classes and shifted faculty and students to online teaching and learning. This move was intended to allow flexibility in teaching and learning while maintaining high learning outcomes and expectations. While many institutions were unprepared to quickly shift to virtual learning (VL), the Higher Colleges of Technology (HCT-UAE) was uniquely positioned. This is mainly because of the institution’s existing use of technology, blended learning, e-portfolios, learning management systems, eAssessment, digital library services, intelligent learning systems, digital services, and infrastructure (HCT^4^.^0^, ^2020^). During the transition to full online teaching, HCT offered support to faculty and students by transforming to a Digi-campus and maintaining ongoing communication channels.

Ultimately, HCT’s online courses during the pandemic differed from regularly planned online classes. The success of the new virtual learning environment (VLE), as with traditional learning environments, begins in the classroom. Therefore, research into online learning at HCT was pivotal to understand the obligations and operations of the new approach.

Although HCT focuses on technology use, the sudden transition to VL led to a new array of leading and managing learning and teaching in online communities. Geng et al. (2019) refer to this as a blended learning environment. While VL became the most prominent method of delivery for higher education during the pandemic, it presents specific challenges for students’ effective learning and communicating within the new context. Generally, virtual classroom learning is not without drawbacks, such as the level of instructors’ readiness, social issues with students, and a lack of “real” online community that ensures positive learning outcomes (Fryer & Bovee, 2016; Murphy & Stewart, 2017).

Students caught off-guard by the sudden shift to virtual classrooms encountered challenges engaging in courses that provided community-based experiences to ensure positive social and cognitive interaction among learners and instructors (Early & Lasker, 2018). Efforts to improve these interactions prompted researchers to identify factors to support successful online experiences (Le Roux & Nagel, 2018). The Community of Inquiry (CoI) model was proposed as it examines the quality of VLE (Anderson & Dron, 2012; Swan & Ice, 2010). The framework integrates the student as a social presence, the teacher as a designer and facilitator, and the cognitive as the presence that constructs meaning through sustained communication. The role of CoI in this interpretive phenomenological research, therefore, is to provide insight into the lived experiences of Emirati female students who were forced to adapt to VLE during the pandemic. This research attempts to answer the following three questions:

1. How have cognitive, social and teaching presences influenced Emirati female college students’ perceptions of online classes during the sudden shift to the virtual learning environments?
2. What relationships exist among cognitive, social, and teaching presence and students’ lived experiences during the sudden shift to virtual learning environments?
3. What do Emirati female college students consider as challenges in their new virtual learning environments?

## Review of literature

### Research on virtual learning environments during a crisis

During the COVID-19 crisis, educators needed to ensure students’ ability and willingness to work productively within their new learning environments (Hew et al., 2020). Educators hoped virtual CoIs would maintain a healthy social, teaching presence that positively affected students’ cognitive presence (Hew et al., 2020). Any adverse disruption in education, e-learning, and educational institutions is a general concern among instructors although crisis and educational disruption was a normal occurrence for some countries before the pandemic. For instance, Rhema and Miliszewske (2012) found Libya’s ongoing political crisis created educational setbacks in face-to-face format and resulted in a lack of motivation among teachers and students. Similar situations exist in conflict-torn and under-developed areas such as the Middle East and Africa (Ehsan & Faqiry, 2021; Samarakoon et al., 2017). The use of technology-assisted learning was proposed to reintroduce functionality and advance the presence of e-learning.

Earlier, Friedman and Friedman (2011) suggested online learning as pivotal to manage educational crises resulting from overspending, disengaged students, and low standards. Their recommendation premised an inclusive model that incorporates schools from the elementary to tertiary levels. Inclusivity was important during educational crises to avoid students being disenfranchised. Recently, Huang et al. (2020) highlighted China’s use of open educational practices. The researchers suggest that any VLE during a crisis should allow for flexibility of learning, ensure reliable network infrastructure, utilize friendly learning tools, adopt suitable digital learning resources, facilitate effective online teaching and learning, and, finally, provide supportive services for teachers and students.

Active instructors are important influences on students’ learning success and engagement. During VLE, the instructor’s presence is recognized differently than in a traditional classroom. The mode and level of interaction between students and other systems in VLE can intuit signs of students’ successful development (Akyol et al., 2009; Richardson et al., 2017). The process, therefore, requires a form of engagement with an inimitable level of support and engagement for both users as found in the CoI model. The CoI framework includes consideration for the teaching climate, content, cognitive presence, teaching presence, social presence, and supportive discourse to engender a meaningful online educational experience. This is especially germane in the current context. Healthy and appropriate interaction between social, teaching, and cognitive presences should lead to higher levels of student satisfaction in VLEs (Kozan, 2016; Ma et al., 2017).

### Concept of the virtual environment of inquiry presence

Since VLEs lack face-to-face presence, researchers investigated this concept within VL contexts (Garrison et al., 2010a), the nature of various presences, and how they interact (Rolim et al., 2019). For instance, Shin (2002) focused on social presence in distance learning as students indicated a need to connect to learning resources and supportive online sources. Stodel et al. (2006) also described learning experiences as maintaining certain learning objectives within a social community. Drawing on Garrison et al.’s (2010a) social, teaching, and cognitive presences of CoI, Hew and Cheung (2011) highlight the importance of these roles in students’ learning experiences as they facilitate collaboration and higher-order thinking skills. However, creating a community of investigation and higher-order thinking can be challenging for educators teaching in virtual environments. Often they require more than a social presence to overcome these challenges. To enable VL, educators need to create communities of critical inquiry (Garrison et al., 2001). The proposed CoI framework encompasses practical ways to help students in online communities actively participate and share meaning.

### Community of inquiry framework

The CoI model outlined the main aspects imperative for students’ successful VLE (Shea & Bidjerano, 2009). Researchers validated this three-dimensional pedagogical model when applied to a full VL format (Akyol et al., 2009; Arbaugh et al., 2008; Kanuka et al., 2007; Le Roux & Nagel, 2018). According to Garrison et al. (2001), the three essential dimensions that contribute to powerful VLE in the CoI pedagogical model are cognitive presence, social presence, and teaching presence.

*Cognitive presence* focuses on learners’ development of higher-order thinking skills and construction of meaning as they move through the four cyclical stages of inquiry and sustained reflection (Garrison et al., 2001). The first stage, *triggering*, highlights a dilemma that leads to the second stage, namely, *exploration* of the issue and brainstorming. When students find more information through a collaborative process of exploration and brainstorming, ideas are *integrated* with the third stage to construct meaning and defend this information. This leads to the fourth stage, which is the *resolution* of the initial dilemma through testing and implementation (Garrison et al., 2001). Redmond (2014) identifies reflections as a key aspect of CoI that leads to better cognitive presence.

*Social presence* focuses on students’ development of personal interactions and collaboration within productive virtual social CoIs (Garrison et al., 2001). Lowenthal (2016) defines social presence as individuals’ ability to present themselves as real people through certain communication tools. Garrison et al. (2001) categorize social presence into *emotional (affective)* expressions where learners share values and personal expressions; *open communication*, where they develop mutual awareness and recognition; and *group cohesion*, where they build and sustain a sense of group commitment. Richardson and Swan (2003) found social presence positively affected students’ and instructors’ course satisfaction. Learners who perceive high social presence learn better but to increase social presence among students, DeNoyelles et al. (2014) claim discussions should be designed to provide opportunities to improve student’s communication skills and build shared understanding. Proposed strategies included providing modest feedback, peer facilitation, protocol discussion prompts, and audio feedback. Le Roux and Nagel (2018) characterize these as the effect, open communication, and group cohesion. Generally, implementing discussion strategies that integrate all presences is crucial to support effective CoIs.

Finally, *teaching presence* refers to the instructors’ role and leadership skills during the course (Anderson et al., 2001). Garrison et al. (2001) define teaching presence as designing, facilitating, and directing cognitive and social processes for better learning outcomes. Teaching presence focuses on *course organization and design*, such as planning and setting the curriculum, designing teaching methods and strategies, offering *direct instruction*, such as summaries and content presentation, and *facilitating* discourse, by setting the climate and reinforcing students’ contributions (Anderson et al., 2001; Le Roux & Nagel, 2018).

Swan and Shih (2005) suggest a revision to the CoI model as they perceive the teacher’s presence better determines students’ level of satisfaction than peer interaction. Kenzig (2015) notes, however, that instructors require adequate training to enable an effective shift to VL and suggest best practices to facilitate the transition from traditional classrooms. Ke’s (2010) mixed-method approach found cognitive and social presences in online course design and teaching elements are crucial prerequisites for a successful online experience in higher education. Consequently, they encourage online course designers to consider the relationships between COIs and effective teaching.

Regarding the instructional methods and strategies implemented in VLEs, Joo et al. (2011) investigated structural relationships among the perceived level of presence, usefulness, and ease of use of online learning tools, and students’ satisfaction in Korea. The results indicate that teaching and cognitive presences, and their perceived usefulness and ease of use were important predictors of student satisfaction. Their findings also suggested design and implements for online teaching and learning strategies within a higher education context.

To better understand the *relationships* among the three presences of CoI, Garrison et al. (2010b) explored casual relationships in online and blended learning. They tested two hypotheses: (1) teaching and social presence have a momentous effect on cognitive presence, and (2) teaching presence is perceived to influence social presence. Their results highlighted the essential role of teaching presence to establish and sustain a virtual CoI and the importance of the existence of dynamic relationships among all three.

Armellini and De Stefani (2016) confirmed the strength and interrelatedness in the teaching and cognitive presence in VLEs. In their study of 40 English teachers completing online education courses, they track the role of cognitive, social, and teaching presences in professional development. Social presence, particularly, was found to be an integral part of teaching and cognitive presences, rather than a standalone presence.

Regarding *students’ perception* of VLE, Morris (2011) explore community perceived experience of online learning based on the CoI framework. Using models of successful instructional design and course facilitation techniques, Morris (2011) examined how students perceive these three elements interact to create a rich educational experience. Recently, Duran (2020) explored the lived experiences of online silence during learning, using interview data from 12 graduate students. The analysis reveals silence as a complex and multifaceted phenomenon enacted by participants and received by others. Duran explained that speaking out online was done carefully, sometimes with a partial or fuller voice, as an obligation, or with a sense of spontaneity and connection.

### Bioecological perspective on virtual learning

Bronfenbrenner (2001) perceived human development as the bidirectional interaction between individuals (biological beings) and the interconnected systems around them (ecology). Researchers furthered Bronfenbrenner’s understanding to provide a new perspective of the holistic integration of interpersonal relationships with larger societal, cultural, and political dimensions (Brendtro, 2006; Swick & Williams, 2006). Bronfenbrenner (2001) claimed that solidification of human relationships within supportive environments increases development, learning, and high learning outcomes. This aspect of human development is essential in developing productive VLEs of inquiry that facilitate cognitive, social, and teaching aspects.

Bronfenbrenner (2001) also refers to the environment in which an individual’s development occurs as a chain of interacting systems, as proximal processes occur due to sustained interpersonal interactions (*relationships*) (Ceci, 2006). These proximal processes are bidirectional, in that the ecology changes the individual and vice versa. Productive learning environments require proximal processes (*relationships*) to achieve expected positive learning results (Ceci, 2006; Merçon‐Vargas et al., 2020). Well-designed learning endowments without relationships fail to achieve targeted objectives.

The bioecological perspective includes a process–person–context–time model (PPCT) design that consists of the four main components of the bioecological theory of human development (Lerner, 2005). The *process* is considered as the bidirectional relationship between an individual, other individuals, and their ecology. Positive interactions are powerful in determining the individual’s development. *The person* includes the characteristics of an individual’s development including genetic, physical, psychological, and behavioral development. *The context* of an individual’s development comprises the interacting systems and social characteristics surrounding the individual such as school structures, learning environment, and disciplinary procedures (O’Toole et al., 2014). *Time* is represented in this research as the current work with recognition of educational transitions as critical times in the lives of students and their families (O’Toole et al., 2014). The bioecological perspective proposes learning outcomes will improve by supporting an individual’s interactions and environments (Bronfenbrenner, 2001), including VL (Smith, 2011).

Smith (2011) contends that applying the eco-biological perspective on learning requires a focus on the *context* of learning to determine positive learning outcomes. That is, desired learning experiences emerge through bidirectional interactions between the individual and the wider surrounding systems. The primary focus is on the incorporation of three types of relationships (teacher-learner, learner-learner, and learner–other individuals) that are essential in supporting VLE’s effectiveness (Smith, 2011).

The VLE is a result of the diverse interaction of systems that affect learning. When each system positively supports the other, salient learning results are imminent. Therefore, students’ positive experiences within the VL context include all various aspects and should drive positive experiences. During the expansion of VL and teaching, models and positive learning experiences are essential. The integration of an individual’s healthy context with successful VL practices (bidirectional relationships) will lead to positive learning experiences.

### Relationship between the bioecological perspective and the CoI framework

Beaver et al. (2009) and Rutter (2006) suggest that genetic makeup controls not only human traits but also the genetic messages that interact with environmental experiences to predict learning outcomes. This means that genetic makeup and ecological experiences interact to predict an individual’s outcomes such as VLEs (Wainschtein et al., 2019). The genetic blueprint consists of potential learning; however, the processes of actualizing genetic potential are found in relationships (Wainschtein et al., 2019). Therefore, learning is a result of the interactions between individuals, the ecology, and the context of learning. Genes have a great influence on outcomes development; however, most outcomes such as learning are determined by interactions and relationships between individuals and the ecology of learning (Tucker-Drob et al., 2013).

The ecological perspective of teaching and learning in VLEs requires focusing on the tools as well as the context of teaching and learning. In the CoI model, these tools are represented via different strategies that are required to successfully enable each presence to affect students’ learning context, especially the relationships between students and teachers (teaching and social presence) and students and other students (social), to result in positive cognitive outcomes (cognitive presence). This shows learners are active participants in the ecology of learning to create their learning through bidirectional interactions. Learning can occur due to the interaction of the individual with the wider community or system, such as the learner and the new virtual CoIs. In VLEs, the combination of personal relationships between the learner and the instructor, and the learner and other learners can lead to positive cognitive outcomes.

From the ecological perspective, many interacting influences such as social environment, teaching and design, and cognitive presence affect students’ learning, as presented in the CoI framework. When each aspect of the dimensions of each system positively interacts and supports each other, the learner can achieve better learning experiences. Students are active participants in these interacting systems of VLEs, and their traits affect other learners as well as the system as a whole. Generally, active and positive collaboration within virtual teaching and learning environments can motivate students toward better collaboration with different dimensions of the new virtual learning system for better learning experiences.

Under the ecological perspective and guided by the CoI framework, timely access to online materials in the new learning environment is insufficient. Rather, instructors need to contextualize these materials to adjust their teaching to the new virtual environment. Moreover, teaching methodologies and strategies in a VLE drive student engagement supported with assessment and feedback strategies (Johnson & Aragon, 2003). From the ecological perspective, this is supported by developing the quality of bidirectional relationships in students’ experiences.

## Methods

### Research design

This study was guided by interpretive phenomenology. The primary purpose was to extract participants’ lived experiences to arrive at a better understanding (Creswell, 2014; Neuman, 2011). Interpretive phenomenology explored questions about daily experiences (Van Manen, 2016).

I employed a convergent parallel mixed method design, consisting of a survey and interviews. I used the Husserlian method of bracketing (Van Mannen, 2016), to ensure validity in data collection and analysis (Ahern, 1999). Bracketing ensured the researcher’s own experiences and beliefs did not influence participants’ understanding of the phenomenon (Carpenter, 2007). The role of the researcher was important to examine the quality of the new VLEs leading to the three-dimensional CoI from the perspective of bioecological theory. All phenomena included various patterns that interacted to create a system in the lived context, and the common patterns provided deeper insight into the given phenomenon (Drack, 2009). Emirati female college students’ experiences allowed for description and interpretation of their learning experiences, how they constructed their worlds, and what meanings they attributed to their experiences (Koro-Ljungberg et al., 2009; Merriam, 2009).

Since this research also considers the bioecological theory of learning, the qualitative approach allowed for an emphasis on the context that included emergent data from the Emirati context of VL (Rossman & Rallis, 2003). The perspective of bioecological theory led to a focus on the context since the environment and climate in which Emirati college students’ lives were integral to understanding their lived experiences in the new VLEs.

### Participants, sampling and data collection

Participants were Year 1 female college students enrolled in a teacher education program at the HCT aged between 18 and 22, and married (*n* = 150) with family responsibilities or single (*n* = 200) with responsibilities for families and siblings.

At the quantitative phase, convenience sampling was used to select participants, explore their VLE, and learn about their challenges and opportunities while studying at HCT. According to Heiman (2008), convenience sampling allows access to conveniently available participants and is a common sampling process in education. Here, all participants shared similar lived experiences and were exposed to blended learning.

For the qualitative phase, we employed purposive sampling (Creswell, 2014). Selected participants were sent follow-up emails and invited to volunteer for this second stage. Ten (10) participants responded affirmatively and semi-structured interviews were conducted along with classroom observations. Creswell (2014) noted three to ten was a typical number of participants for phenomenological studies.

### Data collection

Ethical approval was received from the IRB of the HCT, Abu Dhabi, Al Ain campus. A pilot study was conducted. The data collection instrument for the survey was based on the CoI framework. Permission was received from Garrison via email (April 2020), and the instrument was assessed for fit by HCT instructors who also validated its use for the HCT community. The instrument was found to be valid, reliable, and an efficient measure of the dimensions of social, cognitive, and teaching presence (Akyol et al., 2009; Arbaugh et al., 2008; Kanuka et al., 2007). The English version of the instrument was translated into Arabic and found to be reliable in scale. Interview questions and journal prompts were selected and written based on previous surveys of CoI from the literature (Akyol et al., 2009; Arbaugh et al., 2008; Kanuka et al., 2007). To ensure consistency between the quantitative and qualitative tools, the package was sent to three faculty members to check the appropriateness of the links and ensure consistency with the CoI model and the current Emirati VLE. Triangulation was enabled through descriptive data of the survey, interviews, participant observations, journal entries, and peer-reviewed literature.

Two students who were not a part of the study volunteered for the pilot. Both students were sent an email with the protocol and asked to sign the accompanying consent form and return it via email. Feedback indicated more in-depth responses would be provided if the interviews were administered in Arabic. Further, it was suggested social presence should be approached from a different aspect because participants had met each other during face-to-face learning and the sudden shift to VL occurred after two months on campus. The volunteers also highlighted the need to clarify some questions and link these to the UAE context during the actual interview while retaining the main questions for validity purposes.

The questionnaires were then distributed to approximately 350 Emirati female students in the program. All students responded, representing a 100% return rate. For the follow-up qualitative phase, ten participants were interviewed for general information and reflections, until I reached data saturation (Charmaz, 2008). Interviews were conducted for a month during the second term because participants’ experiences were current and they were more accessible. Each interview lasted 45 min.

### Data analysis

The CoI survey instrument was analyzed using descriptive statistics using Excel. Multiple choice questions for 6–7, and 22 were graded as (0) Yes and (1) No; questions 1–5, 9, 11, 13, 16–17, 19–20 were graded as (0) Yes, (1) No (2) Somehow; questions 8, 10, 12, 14–15, 18, 21, 23–34 were graded as (0) Yes, (1) No, (2) Somehow, (3) I don’t know. The comments regarding students’ challenges working in the online environment, the role of family, culture, and tradition, and the role of the college were grouped under Qs 35–37 which invited open-ended responses. Analyses were performed with simple statistics. The most positive responses received were Q4-clarity of due dates and deadlines (95%); Q6-instructor’s explanation of the subject in a way that enabled learning (99%); Q7-instructor’s provision of activities that kept students engaged (99%), Q9-instructor encouraging students to explore new topics related to the subject (99%); Q13-timely feedback (96%); Q20-all students felt able to disagree with peers online (100%). The most negatives responses were Q19-students being comfortable dealing with other persons online (97%); Q22-the belief that online classes improved their work skills and collaboration (98%).

The qualitative data were analyzed using thematic analysis (Clarke & Braun, 2014). The recorded interviews were transcribed and then read and re-read for inaccuracies. I uploaded the files in Nvivo and then coded the analysis based on the three general themes following the framework: Teaching, cognitive and social presence. I tried to find similarities among the ten transcripts to elicit sub-themes. Based on the data produced, I matched these with my observations of students’ interactions in the classroom. For example, for *Teachers Presence*, I looked at students’ active involvement in class such as their willingness to answer questions or whether they paid attention. This impacted *Facilitation* since a quiet student suggested non-involvement. I invited two faculty members of the department to recheck my data. They found a few inconsistencies and I changed those before I presented the report to two interviewees to check and ensure I had not misrepresented their views.

## Results

Eleven main themes emerged from the three presences (see Table 1). These are detailed in the following sections.

**Table 1:** Themes and Sub-themes of Emirati female college students’ experiences

Processes	Main themes	Sample of coded text
Teaching presence	Course design and organization	When we changed to virtual learning, the syllabus was modified
	Teacher facilitation	The teacher provided us with an opportunity to go in groups
	Direct instructions	The repetition [of instructions] is because sometimes we don’t start the instructions at once, and so it has to be repeated
Cognitive presence	Development of problem-solving skills	From the beginning of the term and you assign us scenarios, reflections, and things to be solved in the classroom
	Exploration	The teacher gave us the chance to brainstorm about issues, find different solutions and keep brainstorming until we find good solutions
	Integration	I can link information from different resources to answer a certain problem
	Resolution	I like this application as it helps me in my future with classes
Social presence	Affective presence	Yes absolutely, having me in different groups helped me to know more students especially the high achieving students
	Enabler	After a topic is explained, we are immediately given a task, provided instructions, and sent in groups
	Cohesion	We are provided with different activities to be completed in groups
	Associated benefits	I can talk to my teacher freely and tell her my disagreement I feel confident because they trust me and trust my knowledge

### Student perceptions of the three community of inquiry presences

**Fig. 1:**
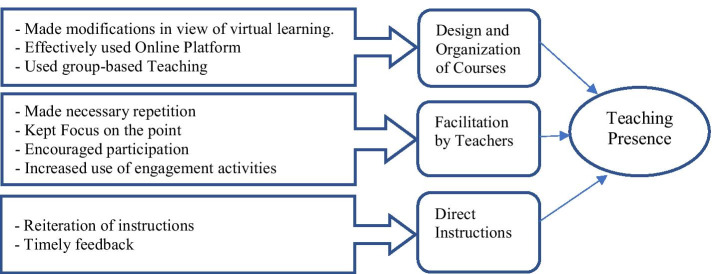
Structure of students’ perceptions about teaching presence in virtual learning environments

Participants’ interviews revealed three themes regarding teaching presence in the VLE (see Fig. 1). Theme 1: *Course design and organization* The sudden change in the teaching environment forced curriculum changes. One participant described: *When we changed to virtual learning, the syllabus was modified. The teacher communicated this to us, including modified tasks and due dates. However, all topics were still included in the course*. Furthermore, teachers used the online environment effectively, and the course content was well-organized.

Theme 2: *Teacher facilitation* Participants noted teachers repeated concepts for clarity. One participant observed: *Whenever there is something that I don’t understand when I ask, I get the answer. She will repeat until I understand*. Teachers also kept students focused. One participant described: *When you discuss something and examples are given, some students laugh. This can take the class in a different direction. You laugh as well, but immediately, you get the students focused and on track*. Teachers also engaged participants with multiple tasks in the VLE. One participant acknowledged this: *The good thing in our class is that we are given more tasks than talk, which is very good for our learning*.

Theme 3: *Teachers’ direct instructions* Teachers reiterated instructions. One participant noted: *A lot of repetition and many activities. For example, repetition is done because sometimes we don’t start the instructions the first time, and it is repeated. However, students who disappear and return require more instructions and repetition*. Participants also acknowledged teachers gave instant feedback. One participant said: *You are given feedback through a shared screen, so we can model and scaffold our learning. Yes, this helps us a lot to easily complete our work*.

**Fig. 2:**
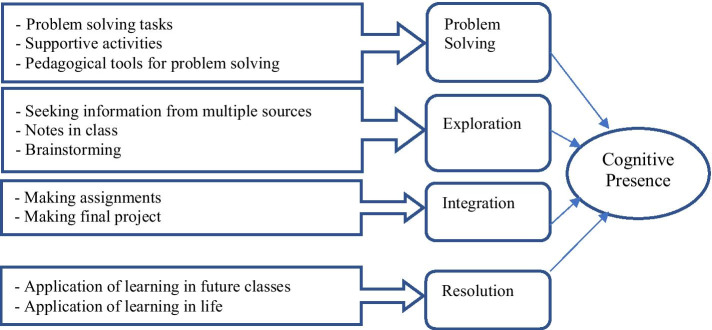
Structure of students’ perceptions about cognitive presence in virtual learning environments

Cognitive presence revealed four themes (see Fig. 2). Theme 1: *Development of problem-solving skills* Teachers provided various activities in classes to develop participants’ problem-solving skills. One participant explained: *A lot of activities were provided to support problem-solving through reflections, brainstorming, and other tasks*.

Theme 2: *Exploration* Teachers encouraged participants to seek information from multiple sources to comprehend concepts and take notes during lectures. A participant acknowledged: *You are always encouraged to take notes and write main points, use the library, visit websites, and watch videos*.

Theme 3: *Integration* Participants recounted being given assignments and projects by teachers in the VLEs to access, gather, perceive and assimilate information from different sources. One participant stated: *I can use different resources to create projects like the toy. When this information is linked to task completion, we are forced to complete, read, explore, link information and create the final product.*

Theme 4: *Resolution* Teachers engaged students via VL to apply learning in their lives. One participant mentioned: *In addition, I learned how to be on time and organized, which is very important for real life.* Thus, VL helped participants to be responsible and organized.

**Fig. 3:**
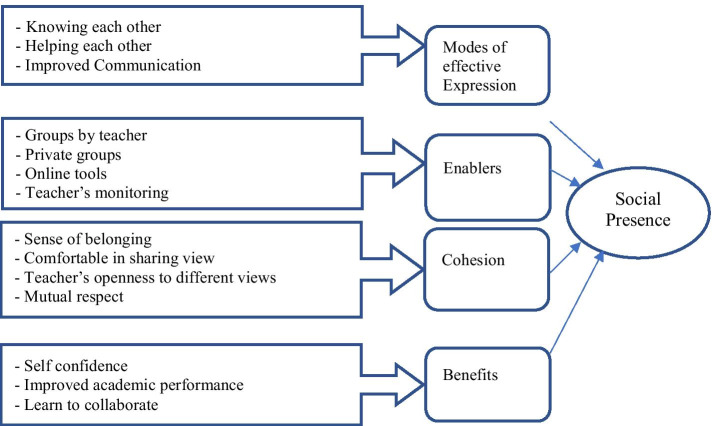
Structure of students’ perceptions about social presence influence in virtual learning environments

Participants’ perceptions of the influence of VLEs on their social presence identified four themes (see Fig. 3). Theme 1: *Affective expression* VL brought participants closer, and they grew familiar due to increased interaction and group work. One participant stated: *Honestly, during times on campus, I wasn’t very close to students in my class maybe because I am one year ahead of them. However, online learning enabled me to be close to them*. Increased interaction, group work, and peer assistance via VL forums also improved participants’ communication. One participant said: *This helped me to communicate a lot with [other] students. This helped me to raise my academic achievement by improving my performance in completing tasks*.

Theme 2: *Enablers* These initiatives facilitated a social presence among participants. The first enabler was a teacher assigned student groups that allowed students to discuss class topics among themselves. For instance, a participant mentioned: *After you explain a topic, you immediately give us a task, provide instructions and send us to groups. There, we feel that we are forced to discuss information with each other. Then, I discuss my findings with other students in the group to present in the main room*. Participants also established informal, private groups, as follows: *We exchanged our numbers and talked privately, supported each other in completing assignments and tasks, and explained difficult points for each other. We also created private groups*.

Theme 3: *Cohesion* Participants developed a sense of belonging working in groups. One student participant stated: *I feel like students do care. For example, if a student has an issue with her laptop, other students come and inform you because they care. They respect each other*. Moreover, participants felt comfortable talking to their teachers. One participant expressed: *I can talk to my teacher freely and tell her about my disagreements. I feel safe and comfortable*. Teachers were observed to be very supportive in the VLEs, as confirmed by a participant: *I feel comfortable and confident when I share a different point of view. The teacher is supportive and creates a comfortable environment where everyone can share her point of view*.

Theme 4: *Benefits of using the VLEs* Participants reported improved confidence in class. For example, one participant noted: *This improves my self-confidence, and I want my voice to be heard as I am one of those who attended the whole lesson*. There were other benefits as noted: *This improved the social interaction between us. This taught us to collaborate.* During VL students’ increased use of various forums, their academic performance also improved due to social interaction, improved confidence and trust, and care for other students.

### Challenges with virtual learning environments

**Fig. 4:**
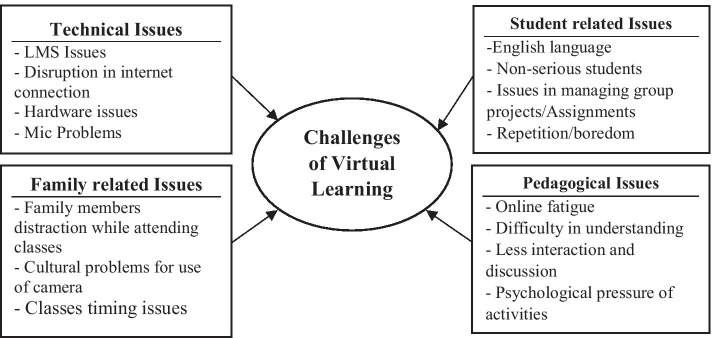
Challenges associated with virtual learning environments

Participants identified various challenges with the VLE (see Fig. 4). These were classified into four categories. Category 1: *Technical or infrastructural issues* associated with VL implementation from home. One participant explained: *Some people do not have many rooms in their homes to have every person in one room. Because I am married, sometimes I can’t study or discuss while my husband is in the room, and because I live in his family’s home, I can’t find a free space for studying, to talk freely, and use the cameras.*

Category 2: Associated challenges with VLEs. Participants mentioned issues such as *language barrier*: *Discussion, as you mentioned, is not deep and fruitful sometimes because they [other students] heard only half of the questions as they were away, maybe because of the language barrier.* Another issue was students’ *lack of interest* in their studies: *The issue is with lazy students. They open the session and then sleep. Staying up late at night prevents students from being interested in early morning lessons.* Participants also mentioned problems while working in groups with unserious students.

Category 3: *Students’ behavior and attitudes* Some participants showed irresponsible behavior during class: *Students who do not focus, get distracted easily and keep asking the same question over and over again. The teacher wastes time repeating instructions because of their irresponsible attitude.* Some chose to be silent during sessions and encouraged others to do the same: *In online classes, she wants students to speak, but they are reluctant so they go silent sometimes and encourage us to do so too*.

Category 4: *Family-related problems* Some families objected to the use of cameras in virtual classes. One participant explained: *I think some students hesitate to use the camera and microphone because they don’t want other students to record them due to cultural consequences.* Siblings at home also posed a distraction. One participant said: *I wish my mother would help me better in terms of supporting my siblings so they are not so distracting. I wish she would help me wake and prepare them for classes*. Furthermore, some participants faced difficulties attending class on time. One participant described: In *the beginning, I lost focus because the system at home had changed. We stay up late, and we have to wake early.* Thus, it was inconvenient for participants to attend early hour classes in the virtual environments.

## Discussion, conclusions, and limitations

**Fig. 5:**
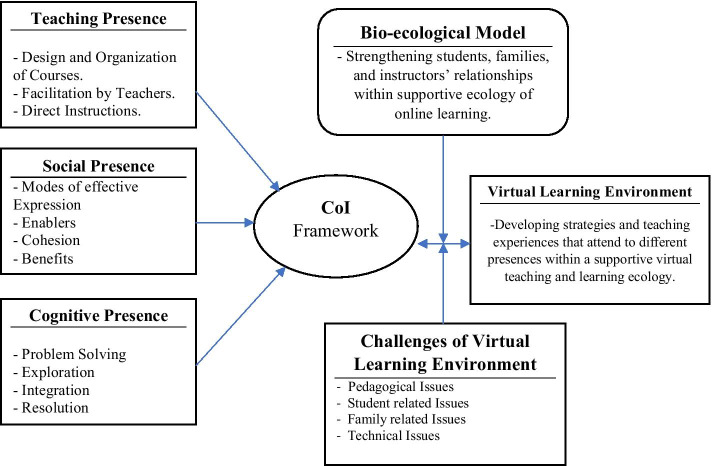
Relationships among the CoI framework, bioecological model, and virtual learning environments

This study explored the perceptions of female Emirati college students concerning VLEs at the HCT, following the sudden shift to online classes, and drew on the CoI framework and bioecological perspective (see Fig. 5). Three research questions sought to understand how cognitive, social, and teaching presences influenced these students’ perception of online classes during this shift; the existence of any resultant relationships; and what they perceived to be challenging in their VLE.

The results showed students identified a need for modifications in the course designs and organization of teaching presence. Teacher’s support and facilitation also encouraged online participation and the use of various activities introduced to encourage engagement. Students were also satisfied when teachers clarified instructions and provided timely feedback on the graded activities thus supporting their capabilities in the VLE (Kozan, 2016; Ma et al., 2017).

Cognitive presence demonstrated how learners understood, constructed, and comprehended meanings in a sustained dialogue (Garrison et al., 1999). In the analysis of this dimension, students reiterated a need for teachers to use different pedagogical tools to teach problem-solving skills. Students should be encouraged to explore and brainstorm different ideas through group projects and group task assignments (Le Roux & Nigel, 2018). Students also indicated their knowledge should support them to solve real-life, practical problems.

In their perception of social presence in VLEs, students indicated the need to use multiple modes of effective expressions such as frequent communication and interactive group formations. Participants suggested formal groups assigned by teachers, informal groups, and participation in learning forums, which should be monitored by teachers (Merçon‐Vargas et al., 2020).

An effective expression can build group cohesion through mutual respect, sharing of ideas, teachers’ encouragement, and a sense of belonging, which helps to establish an effective social presence (Garrison et al., 2010b). These initiatives increased students’ self-confidence, and they learned to collaborate, which resulted in improved academic performance.

The sudden shift to VL posed various challenges to students. One important finding was the ongoing technological challenges despite HCT’s e-focus. Technical challenges included poor internet connection and a lack of supportive information technology infrastructure, which was supported in the literature (see Cullinan et al., 2021). Student issues included English language barriers (Subekti, 2021), non-interest in the virtual classes, problems in group formation, and fatigue using the technology (Hwang et al., 2021). Family and cultural issues also hindered students’ performance in the virtual environment (e.g., restrictions on on-camera use and distractions in the home environment).

Although online education has flourished in the last decade, its real importance has magnified since the pandemic. The bioecological perspective identifies learners as active participants in interactive learning environments (Smith, 2011). Figure 5 outlined the bioecological model integration with the CoI, showing the influence on students’ engagement and social presence and their enhanced ability to respond to a rapidly changing teaching and learning environment during a crisis.

There were several limitations. First, convenience sampling represents a limited population and cannot be generalized. Second, the study drew from only one department of the university and investigated only one gender. And finally, the phenomenological framework is culturally derived which also has implications for generalizability although the information can be important for other disciplines such as gender studies.

### Recommendations

Based on the findings of this study, the following are recommendations for Emirati educational institutions with a focus on the contextual effects of the CoI framework and ecological perspective.

- In VLEs, teaching pedagogy should be reinvigorated. Class activities should be less complicated, and students should not be overloaded with assignments and projects.
- Teachers should adopt multiple techniques to explain course topics to students and ensure they learn the concepts. Practical examples should accompany concepts.
- Classes should be more interactive (i.e., not just lectures).
- Students should be given opportunities to speak and participate in classes to further develop their interests. (Students indicated teachers muted their microphones).
- Teachers should conduct end-of-class assessments.
- Students should receive timely feedback on their learning curves and areas requiring improvement.
- Educational institutions should establish policies about camera use. Students need to turn their cameras on during online classes so that teachers can monitor attendance, moderate discussion boards, and conduct learning activities. (Students reported parental refusal to allow camera use because other students share screenshots on social media). Educational institutions should draft effective policies and ensure strict monitoring of online learning forums to avoid such incidences.
- Students should be provided adequate support from family members, who should be educated on the requirements of VL.
- Policymakers should ensure that classes are offered in both the local language and English to increase understanding of concepts articulated in English.
- State-of-the-art technology should be used to manage learning management systems and facilitate the seamless implementation of classes in VLEs. Since technology is the backbone of VL, students should be provided with high-speed internet at home and teachers should have similar access at their institutions.
- Further research should be conducted in a similar context to examine design strategies and learning materials that take into account cognitive, social, and teacher presences.

## Data Availability

Data is available from the corresponding author upon reasonable request.

## Abbreviations

CoI: Community of inquiry
COVID (19): Coronavirus
HCT: Higher colleges of technology
VL: Virtual learning
VLE: Virtual learning environment
